# Sacroiliac Indicis Increase the Specificity of Bone Scintigraphy in the Diagnosis of Sacroiliitis

**DOI:** 10.4274/mirt.40427

**Published:** 2015-02-15

**Authors:** Zehra Pınar Koç, Arzu Kin Cengiz, Funda Aydın, Nehir Samancı, Veli Yazısız, Süleyman Serdar Koca, Binnur Karayalçın

**Affiliations:** 1 Fırat University Faculty of Medicine Hospital, Clinic of Nuclear Medicine, Elazığ, Turkey; 2 Adnan Menderes University Faculty of Medicine Hospital, Clinic of Nuclear Medicine, Aydın, Turkey; 3 Akdeniz University Faculty of Medicine Hospital, Clinic of Nuclear Medicine, Antalya, Turkey; 4 Akdeniz University Faculty of Medicine Hospital, Clinic of Physical Therapy and Rehabilitation, Antalya, Turkey; 5 Akdeniz University Faculty of Medicine Hospital, Clinic of Rheumatology, Antalya, Turkey; 6 Fırat University Faculty of Medicine Hospital, Clinic of Rheumatology, Elazığ, Turkey

**Keywords:** Sacroiliitis, scintigraphy, indexes

## Abstract

**Objective:** Bone scintigraphy is a highly sensitive method in the evaluation of sacroiliitis. Aim of this study is firstly to evaluate interobserver variation of partial and whole sacroiliac indicis, secondly investigation of clinical importance of these indicis in the diagnosis of sacroiliitis.

**Methods:** Fourty-six subjects (24 female: 35.4±11.9; 22 male: 43.1±12.4) without sacroiliitis 45 subjects with low back pain (33 female: 43.3±11.5, 11 male: 35.5±17.2) were included in the study. For right (R) and left (L) whole indices (WSI) irregular region of interest (ROI), for partial indices superior (S) and inferior (I) rectangular ROI were used. For background activity, rectangular ROI was drawn from the sacral region. Indices were calculated from ratio of average counts of sacroiliac and background regions. Two independent observers calculated sacroiliac indices. Interobserver agreement was evaluated by Pearson analysis.

**Results:** There was no significant interobserver difference (p>0.05). Significant correlation existed between all calculated indices. Among 45 patients with suspicion of sacroiliitis 15 had final diagnosis of sacroiliitis and all of the Tc-99m methilenediphosphonate planar and SPECT bone scintigraphy results of these patients were concordant with sacroiliitis. There were 8 false positive results in other 30 patients. Seven of these eight patients had normal index values. If the scintigraphy would be evaluated in conjuction with indicis the specificity would increase from 73% to 97% but sensitivity decreases from 100% to 80%. There was significant correlation between the observers calculated indicis (p<0.001).

**Conclusion:** Superior and inferior sacroiliac index values can be used with confidence. If we use sacroiliac index values to confirm positive results; index values can increase the specificity of bone scintigraphy.

## Abstract (tr)

**Amaç:** Kemik sintigrafisi sakroiliit değerlendirmesinde kullanılan yüksek sensitiviteye sahip bir metoddur. Bu çalışmanın amacı, ilk olarak parsiyel ve total sakroiliak indekslerin değerlendiriciler arası değişkenliğini değerlendirmek ve ikinci olarak bu indekslerin sakroiliit tanısındaki önemini belirlemektir.

**Yöntem:** Çalışmaya, bel ağrısı ile başvuran 45 hasta (yaş ortalaması 43,3±11,5 yıl olan 33 kadın; 35,5±17,2 yıl olan 11 erkek) ile bel ağrısı olmayan 46 kontrol (yaş ortalaması 35,4±11,9 yıl olan 24 kadın; 43,1±12,4 yıl olan 22 erkek) dahil edildi. Sağ (Sğ) ve sol (Sl) total sakroiliak eklem (TSI) için düzensiz ilgi alanı (ROI), üst (Ü) ve alt (A) kısımlar için dikdörtgen ROI kullanıldı. Background aktivite için sakral bölgeden dikdörtgen ROI kullanıldı. İndeksler sakroiliak ve zemin aktivite ilgi alanlarındaki ortalama sayımlar oranlanarak hesaplandı. İndeksler iki bağımsız gözlemci tarafından hesaplandı. Gözlemciler arası uyum Pearson test ile değerlendirildi.

**Bulgular:** Değerlendiriciler arası anlamlı fark yoktu (p>0,05). Tüm hesaplanan indeksler için korelasyon mevcuttu. Sakroiliit şüphesi ile çalışmaya dahil edilen 45 hastanın 15’inin tanısı takipte sakroiliit olarak doğrulandı. Bu 15 hastanın hepsinin Tc-99m kemik planar ve SPECT çalışması sakroiliit ile uyumluydu. Diğer 30 hastanın 8’inde sintigrafi yanlış pozitif olarak sakroiliit ile uyumluydu. Ancak bu 8 hastanın 7’sinin indeks değerleri normaldi. Sintigrafinin spesifitesi indeksler ile beraber değerlendirildiğinde %73’ten %97’ye yükseldi, ancak sensitivitesi %100’den %80’e geriledi. Gözlemciler arasında indeksler açısından anlamlı bir uyum vardı (p<0,001).

**Sonuç:** Bu bulgular üst ve alt sakroiliak indekslerin sakroiliitin tanısında güvenle kullanılabileceğini göstermektedir. Sakroiliak indeks değerleri sintigrafide sakroiliit tespit edilen hastalarda kullanıldığında sintigrafinin spesifitesini yükseltmektedir.

## INTRODUCTION

Low back pain is a common complaint in the public. Differential diagnosis of low back pain might be difficult, since it has a wide spectrum of etiologic causes and clinical differences in seronegative and sacroiliitis associated arthropathies. Physical examination represents pain in sacroiliac joint projection after provocation maneuvers, as well as spondylarthropathies associated findings including peripheral arthritis, psoriatic lesions and uveitis. Plain graphs might give an idea about the presence and degree of sacroiliitis by showing sclerosis and ankylosis in the sacroiliac joints.

Radionuclide imaging should be a second line imaging method which provides information regarding both sacroiliac joints and whole body if necessary. Bone scintigraphy is a sensitive, easy and cost effective imaging method in the diagnosis of sacroiliitis. Since there is physiological uptake in the sacroiliac joints it is hampered by this drawback as a diagnostic method and additive methods have been introduced in order to improve diagnostic power of bone scintigraphy. Combination of Tc-99m methylene diphosphonate (MDP) bone scintigraphy and Tc-99m nanocolloid bone marrow scintigraphy has showed that Tc-99m nanocolloid scintigraphy can diagnose acute inflammatory sacroiliitis (1). It has been demonstrated that the addition of quantitative methods (comparing the sacroiliac joint activity with background activity like sacrum) to bone scintigraphy has revealed increase in the sensitivity and has pointed the results in agreement with clinical findings (2).

Magnetic resonance imaging (MRI) might show inflammatory changes, in both adults and children, before the plain graphs (3,4). As in bone scintigraphy there are additive methods in MRI like short tau inversion recovery (STIR) imaging which improves diagnostic power of MRI (5). However, MRI is an expensive method and requires long waiting times. However, early diagnosis and early treatment are extremely important in sacroiliitis.

The sensitivity of bone scintigraphy is high; however, its specificity has to be increased in order to improve diagnostic facility. Aim of this study is firstly evaluate importance of bone scintigraphy in diagnosis of sacroiliitis and secondly the contribution of partial and whole indices to diagnostic performance of scintigraphy.

## MATERIALS AND METHODS

Forty-five patients with low back pain and 46 healthy subjects without low back pain were included in the study. The study was conducted between the years 2005 and 2007. Local ethics committee approved the study and the study was carried out according to Helsinki declaration.

Bone Scintigraphy: Tc-99m MDP three phase bone scintigraphy and single-photon emission computed tomography (SPECT) imaging were performed to all the patients. The scintigraphy was performed by a double head gamma camera with parallelhole low energy all purpose collimator (Sopha DST-LXi). Three phase bone scintigraphy from anteroposterior pelvic region was performed. The images in 64x64 matrix 64 frame each 1 sec in dynamic and 256x256 matrix 500 kcount in blood pool and static images in late phase were obtained. Butterworth filter in order 3 and cut-off frequency 0.5 were applied as post-process analysis. Coronal, sagittal and transaxial slices of SPECT images and three phase bone scintigraphy images were interpreted by five experienced nuclear medicine physicians who are aware of index values by visual interpretation. The criterion for positive diagnostic study was increased activity accumulation in sacroiliac joints compared to sacrum activity as background. The final decision of diagnosis was based on both SPECT and planar image results. Separate evaluation was not performed for planar and SPECT images.

**Figure 1:**
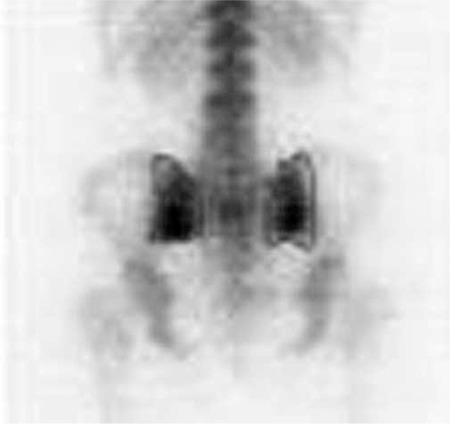
The demonstration of background and whole region of interests

**Figure 2:**
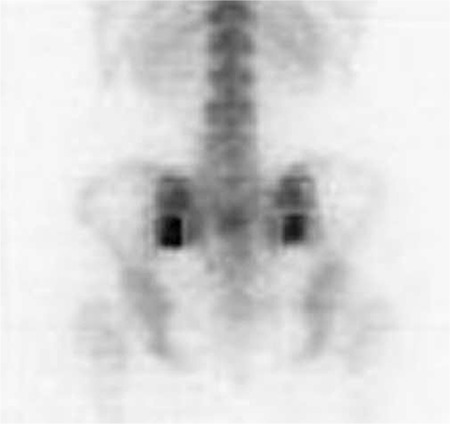
Partial region of interests

Quantification: Irregular region of interest (ROI) for right (R) and left (L) whole sacroiliac indices (WSI) (Figure 1) and rectangular ROI (15x15 pixel) for superior (S) and inferior (I) partial indices (PI) were applied (Figure 2). All of the indices are calculated as the ratio of average counts of whole and partial sacroiliac regions to background region. Index measurements were performed by two independent observers. The values of the patients higher than the maximum index values according to gender shown in Table 1 was considered positive for sacroiliitis. Interpretation of index values was performed according to our own index values obtained from control studies. Additional quantitative analysis for SPECT images was not performed.

The gold standard was accepted as the follow up clinical results and/or additional result of the morphologic imaging method (MR).

The final decision about the sacroiliitis was performed by the clinicians with the combination of the information obtained by anamnesis, physical examination, laboratory results and imaging findings.

**Statistical Analysis**

Statistical analysis was performed using the Statistical Package for the Social Sciences (SPSS 10.0, Chicago, IL, USA). Results were presented as mean ± standard deviation (SD). The agreement between the observers was evaluated by Pearson correlation analysis. Student-t and Chi-square tests were used to compare the parametric and categorical variables, respectively. P values less than 0.05 were considered to be significant.

## RESULTS

The patient group included forty five subjects with low back pain (24 female; mean 34.5±11.9 years old and 21 male; mean 43.1±12.4 years old) and control group consisted of 46 healthy subjects without low back pain (33 female; mean 43.3±11.5 years old and 12 male; 35.5±17.2 years old). There were no significant difference between the mean ages and gender of groups (p>0.05 for both). The sacroiliitis was not observed in any of control subjects by both conventional methods and bone scintigraphy. The index values obtained from control subjects are summarized in Table 1. The index values of both observers were in agreement (p<0.001 for all indices). Significant correlation according to Pearson’s Correlation test existed between both observers for all the whole and partial index values (left whole, left superior, left inferior, right whole, right superior, right inferior) (r=0.8, r=0.7, r=0.9, r=0.8, r=0.9, r=0.8 respectively).

The diagnoses of 45 patients included in this study are summarized in Table 2. Duration of low back pain in patients was 23±23 months. The mean erythrocyte sedimentation rate and C-reactive protein level of patients were 27±22 mm/hour and 1.12±1.87 mg/L, respectively. The follow up period of patients were 12±12 months to assure their diagnosis and gold standard was accepted as follow up and/or additional imaging (MR) results. The low back pain in 15 patients had inflammatory characteristics and FABERE and FADIR maneuvers which are physical examination findings and gives clues about the sacroiliac joint involvement were positive in these patients. All of these patients had sacroiliitis findings in direct radiography, while sacroiliitis in 8 of them were confirmed by MRI.

**Figure 3:**
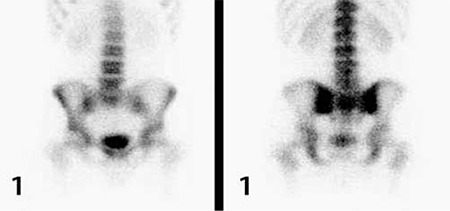
Tc-99m MDP bone scintigraphy images of a 33 year-old male patient with positive right sacroiliitis in direct X-ray; scintigraphy shows bilateral positivity and index values of the patient is increased. Nine year follow up of the patient revealed the diagnosis of sacroiliitis associated with seronegative arthritis

All of the patients who had diagnosis of sacroiliitis based on conventional methods (n=15) also had sacroiliitis according to scintigraphy (Figure 3). Additionally the lateralization of left or right sidesin visual interpretation was in agreement with partial index results (Table 3). Among the patients who had no sacroiliitis according to conventional methods (n=30) 8 had sacroiliitis in visual interpretation of bone scintigraphy; however, among these 8 patients index values of 7 patients were normal (Figure 4, Table 4).

The sensitivity, specificity, positive (PPV) and negative predictive values (NPV) according to these results are given in Table 5. If we combine the information obtained from index values the specificity increases from 73% to 97% and PPV increases from 65% to 92% (Table 5).

## DISCUSSION

Low back pain is an important health problem regarding the associated disabilities and social problems. Seronegative arthropathies which are characterized with sacroiliitis are the most frequent etiology of inflammatory low back pain (6,7,8). Early diagnosis makes biological (tumor necrosis factor alpha blockade) treatment possible which is effective in early phase of spondyloarthropathies (8,9).

Generally the diagnostic methods of sacroiliitis include physical examination, direct radiography, radionuclide methods and morphological imaging like computerized tomography (CT) or MRI. Physical examination or provocation tests are important in diagnosis; however, they cannot provide etiologic information. In a study about multiple direction provocation tests; these physical examination methods improved the predictive values of diagnostic laboratory tests but their discriminative capacity is still poor (10). Direct graphs are important in the presence of positive findings but they do not exclude sacroiliitis (11). Direct graph findings are erosion of bone, alteration of joint space, subchondral sclerosis and ankylosis (12). Direct graph findings occur in 3-7 years, while MRI might show earlier changes (13).

Computerized tomography demonstrates bone changes more effectively than other methods (11). According to an analysis including 1383 CT images, it has been documented that CT is a reliable method and has a good interobserver agreement (14). However, CT documents chronic findings as in direct graphs.

The early changes detected by MRI in patients with sacroiliitis are fluid collection in intra-articular space, proliferation of synovium, bone erosion and bone marrow edema which is the earliest manifestation (15,16,17,18). Marzo-Ortega et al. documented in their three patients that the bone marrow edema finding contributes to osteitis in biopsy results (18). Contrast enhancement increases the diagnostic efficiency of the method and addition of the subtraction methods can also show inflammatory changes (11,16,19). MRI is the best method to show both early and chronic changes; however, correlation of this information with clinical findings is still a subject of discussion (20,21,22,23). It has been shown that only MRI can demonstrate early inflammatory changes but not late phase findings.

Bone scintigraphy has been introduced as a screening tool in diagnosis of sacroiliitis (11). The sensitivity of bone scintigraphy has been found to be 52% in patients without radiographic changes and 66% in patients with grade 2 and 3 sacroiliitis in a study in patients with ankylosing spondylitis (13). The lower sensitivity rates have limited the use of scintigraphy in these previous studies. However those previous studies compared bone scintigraphy with MR and concluded that bone scintigraphy might not have sufficient sensitivity in a specific group of patients. MR is able to detect small changes in the bone marrow before any of the imaging modalities but the only importance in these kinds of subtle changes is in special patient populations like ankylosing spondylitis. The patient population of sacroiliitis consists of symptomatic subjects and among them bone scintigraphy has sufficient ability to demonstrate sacroiliitis. In our study, bone scintigraphy had a high sensitivity (100%) in the diagnosis of sacroiliitis.

It has been known for years that SPECT imaging increases the sensitivity of bone scintigraphy. In this study we also interpreted both planar and SPECT images of patients together. Hanly et al. has reported that MRI is the most sensitive and SPECT is the most specific method in diagnosis of sacroiliitis (15). Thus we performed additional SPECT imaging in every patient with pre-diagnosis of sacroiliitis in our department. However, in spite of additional SPECT imaging the specificity of bone scintigraphy in diagnosis of sacroiliitis was low in our study. However, calculation of index values increased the specificity of scintigraphy (73% versus 97%).

Quantitative methods have been utilized in order to increase diagnostic power of scintigraphy (2,24,25). Recently SPECT/CT analysis has been performed by Cusi et al. in order to investigate patients with peri-partum pain for >2 years and revealed 95% sensitivity and 99% specificity (26). The most important point in evaluation of index values are firstly every laboratory must has own index values and secondly age and sex factors should be considered during the evaluation (2). Zaferiakis et al. have observed differences between different age groups in a homogenous group of young male population (27). We analyzed male and female patients in different groups and the age distribution of the patients were generally homogenous. Two different observers performed the measurements at different times and were aware of each other with same methodology and results of these observers were in agreement. Since they are anatomically different regions we divided the joints in two parts; superior and inferior. In routine practice we observed that sacroiliitis sometimes occurs in superior or sometimes in the inferior part. Thus we thought that whole estimation of joints can cause an underestimation of regional alterations and we developed this methodology. As a conclusion we observed agreement between lateralization of partial indices and involvement of the joint in sacroiliitis. The limitations of this study are not performing MRI in all the patients, only eight of patients with sacroiliitis had additional MRI.

## CONCLUSION

Bone scintigraphy is a noninvasive and cost effective method and there is a great deal of experience in this field. The high sensitivity and NPV percentages detected in our study may suggest that the method may be used as a screening test. It is a subject of investigation to increase the specificity of bone scintigraphy and probably these investigations will continue for many years. In this study, we showed that with the method we developed for quantification, by an easy process performed in the existing images, especially by re-evaluation of positive visual bone scintigraphy findings, it is possible to increase the specificity of bone scintigraphy.

## Figures and Tables

**Table 1:**
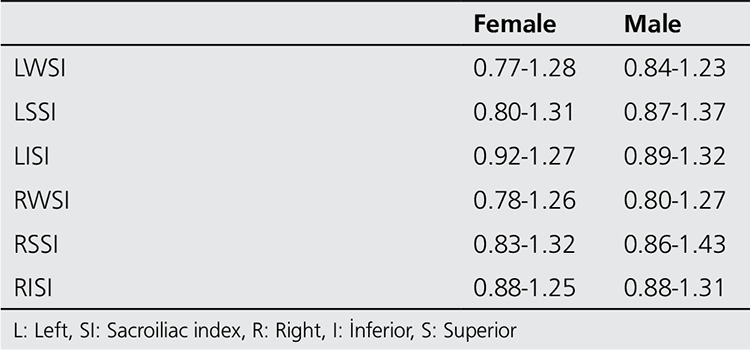
The index values obtained in control group

**Table 2:**
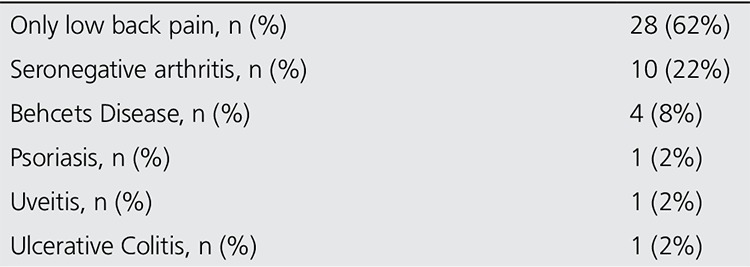
The distribution of patients’ diagnosis

**Table 3:**
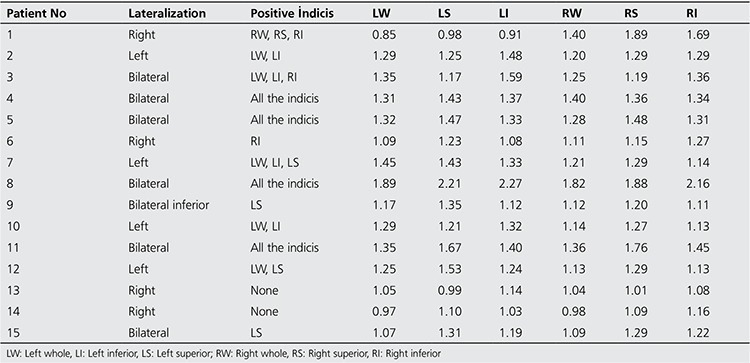
Lateralization of sacroiliitis in positive patients with corresponding partial indicis

**Table 4:**
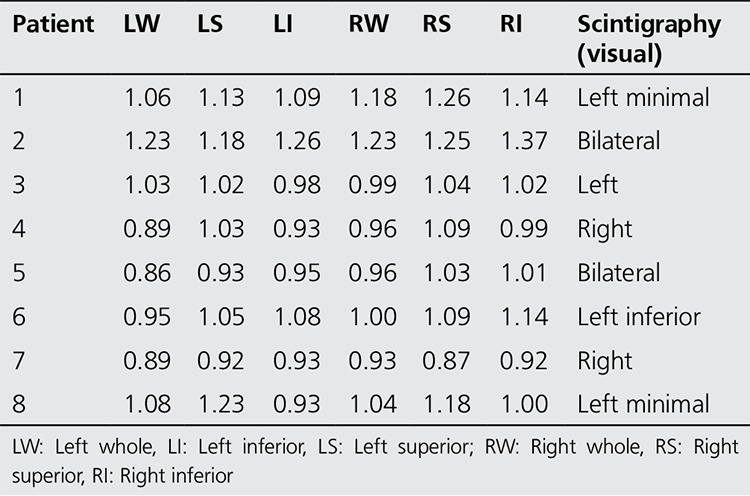
The index values of sintigraphically positive patients without the diagnosis of sacroiliitis

**Table 5:**
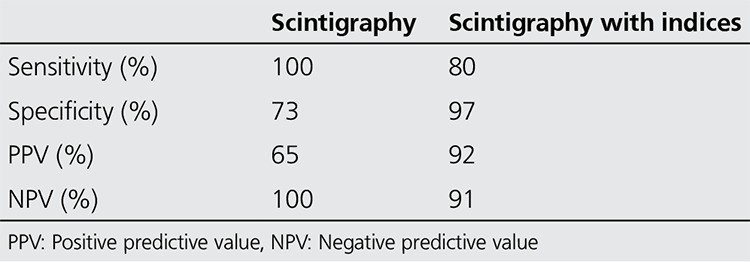
Results of scintigraphy and index values in diagnosis of sacroiliitis

**Figure 4a:**
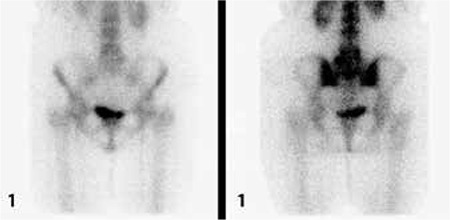
Tc-99m MDP bone scintigraphy images of a patient whose final diagnosis was degenerative changes however scintigraphy was reported as sacroiliitis and index values of the patient were also normal

**Figure 4b:**
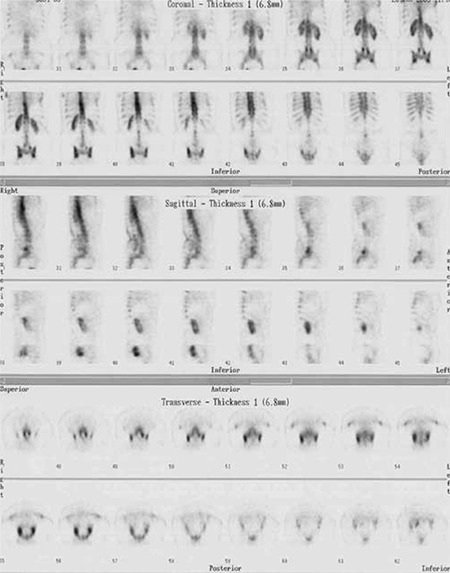
Coronal, sagittal and transaxial slices of SPECT images of the same patient
